# Point-of-Care Ultrasound Diagnosis of Emphysematous Cholecystitis

**DOI:** 10.7759/cureus.104248

**Published:** 2026-02-25

**Authors:** Helena L Kons, Ariana Gorman, Maxwell Thompson, Katherine Griesmer, Samuel L Burleson, David C Pigott, John P Gullett, Luke R Bishop

**Affiliations:** 1 Emergency Medicine, University of Alabama at Birmingham, Birmingham, USA; 2 Emergency Medicine, University of Miami / Jackson Health System, Miami, USA; 3 Emergency Medicine, Oregon Emergency Physicians, Portland, USA

**Keywords:** cholecystitis, computed tomography abdomen, diagnosis, emphysematous cholecystitis, point-of-care ultrasound (pocus), right upper quadrant abdominal pain

## Abstract

Emphysematous cholecystitis (EC) is a form of cholecystitis characterized by the presence of intramural or intraluminal gallbladder gas secondary to infection by gas-forming bacteriaand is considered a surgical emergency. Computed tomography (CT) is considered the gold standard for the diagnosis of EC, but it can result in increased time to diagnosis and may not be readily available in the emergency department (ED). This delay in diagnosis may contribute to increased rates of gallbladder perforation and associated mortality. As emergency medicine door-to-first-provider times continue to increase, point-of-care ultrasonography (POCUS) can facilitate the diagnosis of this serious condition and expedite definitive care. We present a patient with EC diagnosed using POCUS and highlight the utility of POCUS in the evaluation and management of patients with this rare but serious condition. Therefore, early diagnosis and intervention are critical in the management of patients with EC, and POCUS may play an important role in the rapid diagnosis and definitive care of these patients in the ED setting.

## Introduction

Abdominal pain is one of the most common chief complaints for patients presenting to the emergency department (ED) [1]. While acute cholecystitis is a relatively common cause of right upper quadrant (RUQ) pain in the ED, other sources of gallbladder (GB) pathology, such as gangrenous cholecystitis, GB perforation, and emphysematous cholecystitis, represent less common etiologies but may be associated with much higher morbidity and mortality [2]. The timely evaluation of patients in the ED with RUQ pain for whom acute GB pathology is suspected is imperative. While contrasted computed tomography (CT) offers a higher sensitivity with regard to GB pathology, prolonged turnaround times for CT results in the ED setting may lead to significant delays in the timely diagnosis and management of this patient population. Thus, the use of point-of-care ultrasonography (POCUS) can aid in the early detection of disease and should be utilized in patients for whom acute GB pathology is suspected.

## Case presentation

A 69-year-old female with an extensive past medical history, including chronic obstructive pulmonary disease, coronary artery disease, chronic kidney disease, and diabetes, presented to the ED with a two-day history of RUQ abdominal pain accompanied by anorexia and altered mental status. On arrival to the ED, she was mildly tachycardic to a heart rate of 124, with a temperature of 98.4°F, a respiratory rate of 20 breaths per minute, a blood pressure of 110/70 mmHg, and an oxygen saturation of 90% on room air. The patient’s physical exam was notable for significant RUQ tenderness to palpation in addition to a positive Murphy’s sign. Laboratory evaluation revealed a white blood cell count of 7.0 x 10^9^ per liter, alanine transaminase level of 11 units per liter, aspartate aminotransferase level of 21 units per liter, and total bilirubin level of 0.5 milligrams per deciliter (Table 1).

**Table 1:** Patient's laboratory value results with reference ranges.

	Patient lab value	Reference range
WBC	7.00 x 10^3^/cmm	4.00-11.00
Hgb	9.2 gm/dL	11.3-15.2
AST	16 units/L	12-39
ALT	11 units/L	7-52
Total Bili	0.8 mg/dL	0.3-1.4
Alk Phos	69 units/L	37-117

**Figure 1:**
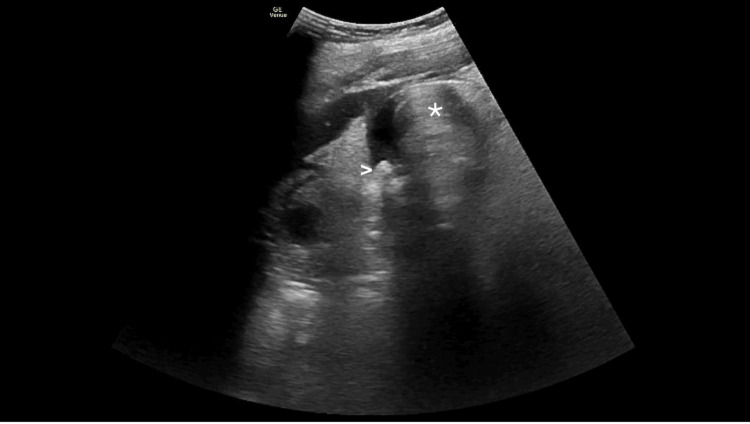
Right upper quadrant point-of-care ultrasonography showing shadowing gallstones (arrowhead) and prominent gas shadowing (*) originating from within the nondependent fundus and obscuring most of the gallbladder and posterior wall.

**Figure 2:**
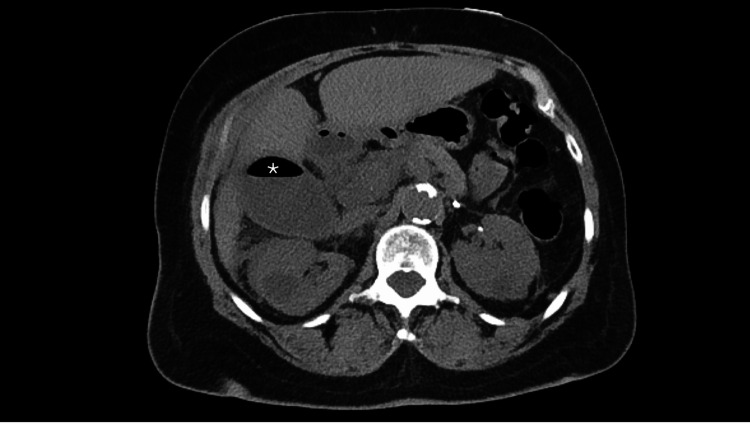
Noncontrasted computed tomography of the abdomen and pelvis demonstrating a large, distended gallbladder with surrounding inflammatory changes and a large collection of gas in within GB (*).

POCUS of the RUQ revealed a distended GB with numerous shadowing stones and air in the fundus (Figure 1). Subsequent CT of the abdomen and pelvis was performed due to the admitting surgeon’s request. This was performed without intravenous contrast due to the patient’s significant chronic kidney disease and glomerular filtration rate of 12 milliliters per minute. CT revealed pericholecystic inflammatory stranding with air in the GB fundus and cystic duct (Figure 2), consistent with acute EC. A comprehensive abdominal US was obtained later in the patient's hospital stay, which revealed findings similar to the POCUS study: gallstones and sludge with pericholecystic fluid and positive sonographic Murphy sign without GB wall thickening. In addition, intraluminal gas was noted that obscured the GB fundus and was consistent with EC.

**Figure 3:**
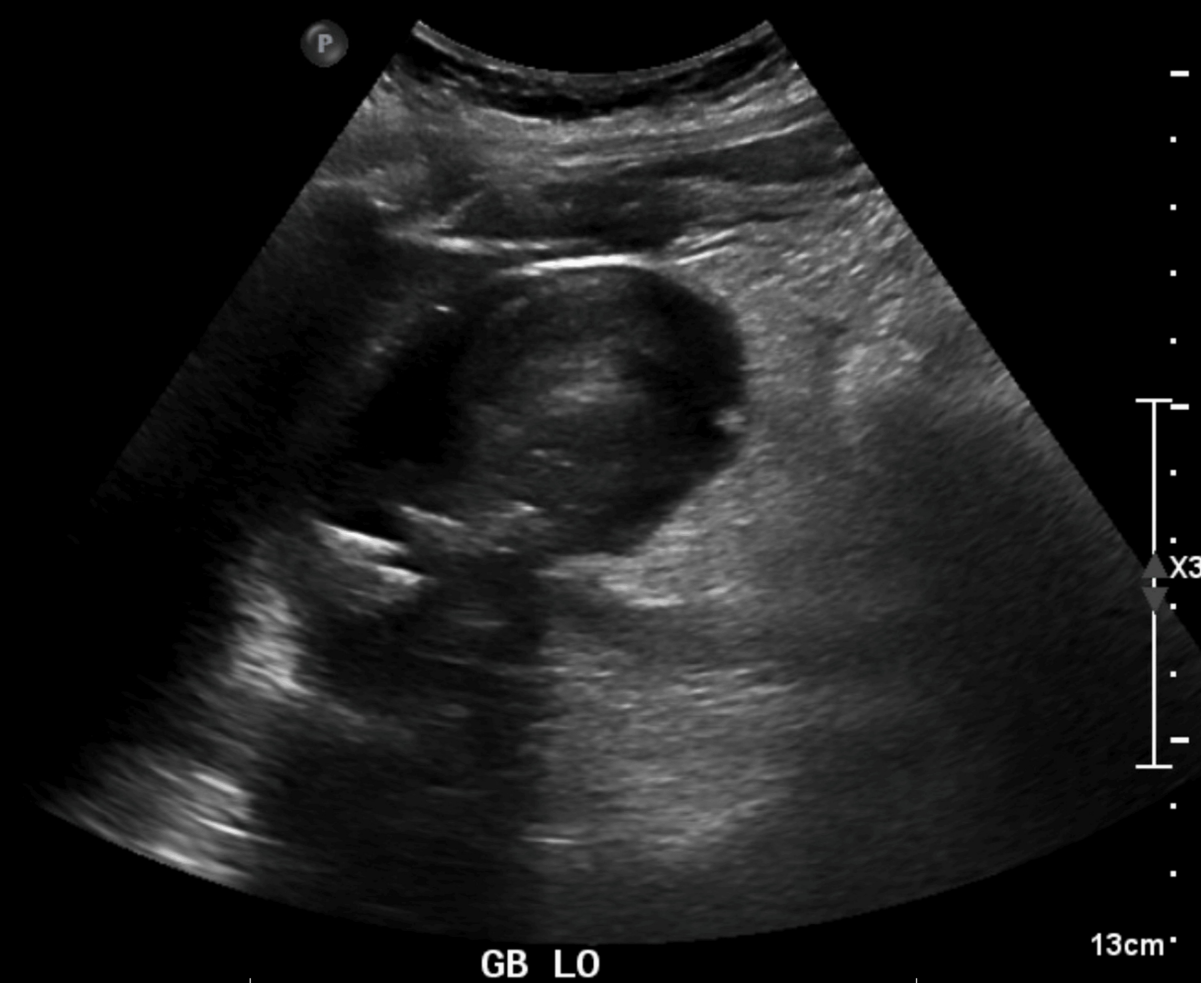
Right upper quadrant ultrasound image from radiologic ultrasound that was also ordered, showing gallstones and air shadowing from the GB wall, read via radiology as emphysematous cholecystitis.

The patient was initiated on broad-spectrum intravenous antibiotics, and gastrointestinal surgery was consulted in the ED. However, the patient was deemed a poor surgical candidate secondary to multiple medical comorbidities, including congestive heart failure, coronary artery disease, chronic kidney disease, and diabetes. Given these conditions, she ultimately underwent cholecystostomy tube placement, and cultures obtained from the GB during this procedure were positive for Clostridium perfringens. She completed a five-day course of intravenous piperacillin/tazobactam with subsequent discharge on hospital day seven with the cholecystostomy tube in place for a total of six weeks. 

## Discussion

EC is a form of acute cholecystitis in which gas-forming bacteria such as *Clostridium* or *Escherichia coli* species infect the GB wall [3,4]. Risk factors associated with EC include male sex, diabetes, age >50 years, previous abdominal surgery, peripheral vascular disease, and immunosuppression. Clinical suspicion in patients with one or more of these risk factors should raise suspicion for EC in the ED [5,6].

EC often presents similarly to uncomplicated acute cholecystitis with right upper quadrant pain, nausea, vomiting, and lab abnormalities, including elevated serum bilirubin levels and leukocytosis [2]. However, laboratory evaluation in these patients may vary, and the mortality risk of EC remains markedly higher than that of acute cholecystitis due to the elevated risk for perforation and gangrene [7]. Thus, prompt diagnosis and treatment in patients with EC is critical. Plain radiographs, POCUS, abdominopelvic CT with contrast, and magnetic resonance imaging (MRI) have all been used to aid in the diagnosis of EC. Due to its high sensitivity in the detection of EC and GB perforation, CT remains the gold standard imaging modality for the diagnosis of acute EC [8]. Despite its higher sensitivity for the detection of EC, the abdominopelvic CT scan is associated with a longer time to diagnosis than bedside POCUS. A recent retrospective analysis from an urban tertiary care center found a median time to CT completion of 108 minutes [9]. This delay in diagnosis associated with CT imaging may contribute to the increased mortality associated with this disease. Despite its lower sensitivity in the diagnosis of acute EC, the use of POCUS has a specificity of 95% in the detection of EC and may aid in earlier detection and treatment of patients with EC and consequently decrease their risk of perforation and mortality [10].

Sonographic imaging results in acute EC vary; certain findings on POCUS offer diagnostic clues in the detection of EC. For example, large volumes of gas in or around the gallbladder can appear as a ring-down artifact, in which echogenic foci from gas bubbles result in a grayish or “dirty” appearance of posterior acoustic shadowing [11,12]. The presence of multiple gas bubbles appears as migrating echogenic foci known as the “effervescent gallbladder” or “champagne sign” [10]. Notably, as is the case with the patient presented above, any detection of intraluminal gas or gas within the gallbladder is also concerning for acute EC. The presence of these imaging findings on POCUS should prompt an expedited CT scan for diagnostic confirmation and immediate surgical consultation. Given its proven use to expedite care in potentially critically ill patients, POCUS remains a valuable diagnostic tool in the early detection of EC in the ED setting. While POCUS does not eliminate the need for an abdominopelvic CT scan in the diagnosis of EC, its ability to aid in the early detection of EC can lead to decreased rates of perforation and improved mortality.

## Conclusions

CT remains the gold standard diagnostic modality for the detection of EC in the ED. However, CT scanning in the ED is associated with increased wait times and may contribute to the increased rates of perforation and higher mortality associated with this disease. Therefore, it is imperative that emergency medicine providers recognize and implement the utility of bedside ultrasonography in making this diagnosis and expediting care.
